# Mutual aid organisations and their role in reducing food insecurity in Chicago’s urban communities during COVID-19

**DOI:** 10.1017/S1368980021003736

**Published:** 2022-01

**Authors:** Saria Lofton, Marjorie Kersten, Shannon D Simonovich, Akilah Martin

**Affiliations:** 1 University of Illinois at Chicago, College of Nursing, 845 S. Damen Ave., Chicago, IL 60612, USA; 2 University of Illinois at Chicago, School of Public Health, Chicago, IL, USA; 3 DePaul University, College of Science and Health, Chicago, IL, USA; 4 AM Root Builders, Chicago, IL, USA

**Keywords:** COVID-19, Food security, Food sovereignty, Mutual aid, Health disparities, Urban environment, Health equity

## Abstract

The COVID-19 pandemic has disproportionately impacted food security and food access in urban communities of colour. Loss of income, often associated with food insecurity, has affected Hispanic, Black, low-wage workers, single mothers and women of colour more than other groups of individuals. Mutual aid organisations have proliferated in response to the COVID-19 pandemic, yet a description of the contributions of these organisations in addressing food insecurity has yet to be described in the literature to date. This article aims to describe the unique role and contributions of mutual aid organisations in addressing food insecurity and food access disparities in Chicago’s communities of colour during the COVID-19 pandemic. Local mutual aid organisations can function as hubs to feed urban communities while reducing food waste and building community. During the pandemic, mutual aid organisations in Chicago have distributed thousands of pounds of food to families and individuals. Mutual aid organisations provide short-term food security while engaging with community members to create a more equitable and sustainable food system. The development of robust mutual aid hubs facilitated unique opportunities for collaboration and expansion of infrastructure that may allow mutual aid organisations to address food access in their communities well into the future.

Across the country, the pandemic has adversely impacted food security and food access in urban communities of colour^(1,2)^. In particular, Black, Hispanic and Asian adults are at the highest risk of being food-insecure during the pandemic^(3,4)^. During the peak of the pandemic, Black food-insecure households were more likely to report they could not afford to purchase more food during the pandemic and Hispanic food-insecure households were more likely to report they are afraid or do not want to go out to purchase food during the pandemic^(5)^. The traditional food system, which includes grocery stores and convenience stores, has failed individuals because food hoarding and disruptions to the food supply left grocery store shelves bare^(6)^, while increased food prices due to price volatility prevented individuals from purchasing the food that they needed^(7,8)^. The emergency food system, which includes food pantries, soup kitchens and food banks, also failed individuals because the emergency food system could not keep up with increased need^(2,3,9)^. Although these food systems disruptions were isolated to the peak of the pandemic and were temporary, they point to cracks in the food system that were not as noticeable before the pandemic.

Previously existing and newly formed mutual aid organisations coalesced to address shortcomings in the food system after recognising the need to address increased rates of food insecurity during the pandemic. Mutual aid organisations, which can be informal or form large national organisations, are rooted in grassroots community organising^(10)^. Mutual aid organisations serve to address gaps and respond to the needs of the community. They provide resources like basic necessities, toiletries, cash assistance and food^(11)^. They rely on the reciprocal exchange of services and resources for mutual benefit within local communities and have historically provided social support through informal networks with limited resources^(12)^. Mutual aid organisations use a strengths-based, holistic approach that emphasises shared power distribution amongst participants^(13)^. Specifically, as it relates to improving food access and food security, mutual aid organisations support the local food system by building social, human and financial capacities, which are foundational in the resilience of food systems^(13)^.

Mutual aid organisations’ approach to addressing food insecurity is distinct from the traditional emergency food system for several reasons. First, mutual aid organisations rely on the expectation of reciprocity, while the traditional emergency food system provides food and monetary resources to purchase food without the expectation of individuals ever giving back^(12)^. In this sense, mutual aid organisations provide resources as a catalyst for personal stability that can then be reinvested into mutual aid organisations and not as a handout. Additionally, mutual aid organisations build community strength and resilience by fostering community collaborations and working at the grassroots level to support one another^(11)^. In contrast, some programmes within the traditional emergency food system are focus on serving the greatest number of individuals and not with strengthening those individuals or communities, so they one day no longer have to rely on emergency food programmes. Finally, mutual aid organisations work to radically reimagine local food systems using a food sovereignty lens^(14)^. Food sovereignty is the right of people to healthy and culturally appropriate food produced using sustainable methods in accordance with the wants, needs and desires of the people themselves^(15)^. Mutual aid organisations align with food sovereignty because they provide community members with the power to define the food they procure and the ways in which food is procured in accordance with the needs of their community. To maintain the local food system, mutual aid organisations established relationships with farmers, producers and consumers. These efforts have generated cooperative agreements between farmers, local producers and community residents^(13,16)^.

In Chicago, approximately forty mutual aid organisations have worked to address food insecurity during the pandemic^(17)^. The organisations created hubs that facilitated collaboration and sharing of food, storage and transportation resources to meet the food needs of communities^(18)^. These community-based efforts have built capacity and developed new relationships to absorb food shocks associated with COVID-19 and future crises. For this commentary, we will provide three examples of effective informal mutual aid organisations developed by community members and are not associated with national or government organisations. These examples were selected because they represent organisations who are addressing food insecurity in various communities in Chicago using different methods.

The Love Fridge Chicago, Rogers Park Food Not Bombs (Rogers Park FNB), and the 19th Ward Mutual Aid are representative of three different mutual aid models. Rogers Park FNB has been engaged in mutual aid for over a decade^(19)^, whereas The Love Fridge Chicago and 19th Ward Mutual Aid were established during the COVID-19 pandemic. All three organisations have missions to address existing inequities in food access and provide community support with slight nuances. The Love Fridge’s mission also incorporates reducing food waste, the belief that food is a right, not a privilege, and that providing food is a way to re-envision safety in communities^(20)^. Rogers Park FNB believes that mutual aid is a way to build relationships and care for one another^(19)^. They also believe that mutual aid is political because it organises against the oppressive systems, often political, that create need^(19)^. 19th Ward Mutual Aid believes in helping neighbours out because everyone has needed help at some point in their life^(21)^.

The Love Fridge Chicago, Rogers Park FNB, and the 19th Ward Mutual Aid all provide food and resources to the Chicago community but use different methods. The Love Fridge operates nineteen community fridges that are stocked with food by volunteers^(20)^. The fridges are free to access by community members during their hours of operation. The Love Fridge models partnership with community-based organisations run by Black and Brown individuals experienced in food security work for mutual benefit. As of November 2021, the Love Fridge had already provided over 10 000 pounds of food for those in need. Rogers Park FNB provides food to communities through community-based distributions, and during the pandemic began food delivery and pop-up distributions to serve over 200 households per week^(22)^. 19th Ward Mutual Aid collaborates with other mutual aid groups and organisations to distribute food, direct financial assistance, resources for internet connection and gift cards to grocery stores for community members in need^(21)^. In total, they have distributed food to 4953 families impacting 17 323 individuals as of June 2021.

Although the long-term impact of COVID-19 is unknown, mutual aid organisations have helped to reimagine a local food system focused on individuals and communities. Mutual aid organisations have engaged community members and collaborated to feed community members when the traditional and emergency food systems could not. They have recruited countless volunteers, formed partnerships with urban farmers, and established new food and funding streams. During the pandemic alone, the three mutual aid organisations highlighted in this work have distributed thousands of pounds of food to tens of thousands of families impacting hundreds of thousands of individuals. They have shifted the food system within Chicago towards one that centres on equity, sovereignty and health.

The necessity of creating a community-driven food system continues beyond COVID-19. Individuals will continue to struggle with food insecurity and with obtaining public benefits within the traditional food system, perpetuating chronic food insecurity. Mutual aid organisations are one component of reimagined food systems. Mutual aid organisations are committed to providing food and other resources to individuals within their reciprocal exchange framework, facilitating self-reliance, strong communities and resilience^(16)^. The growth of mutual aid organisations creates important opportunities for collaboration, support, education and research in urban food communities. One component of mutual aid is advocating for systematic change to address the root cause of disparities, including food insecurity. In doing so, mutual aid is a solution until systematic change is achieved.

Mutual aid organisations rely on continued support from grassroots volunteers, and this support may wane over time. Systematic change to address the root causes of food insecurity is necessary to address food insecurity long term. To foster systematic change, there must be a robust discussion about the role of formal systems, like federal, state and local government, in amplifying the work of those addressing short-term food insecurity in the food system. These systems must consider how to apply a food sovereignty lens when establishing food systems policies and programmes aligned with existing grassroots food security work by groups like mutual aid organisations. Clear federal, state, and local policies, financial resources, and vigorous community support are needed to address the underlying disparities in food insecurity and food access in urban communities. Those interested in beginning mutual aid organisations should build relationships with local leaders to identify community-specific needs and seek guidance from established mutual aid organisations to best address local food access and food insecurity needs of their community. With hope and courage, COVID-19 allows us to collectively re-envision a new food system for the 21^st^-century post-pandemic world. Building on the values exemplified by these mutual aid organisations, a re-envisioned food system can be grounded in local food sovereignty, an abundance of food security, and assurance of access to healthy food for all.
